# Linseed, Baru, and Coconut Oils: NMR-Based Metabolomics, Leukocyte Infiltration Potential In Vivo, and Their Oil Characterization. Are There Still Controversies?

**DOI:** 10.3390/nu14061161

**Published:** 2022-03-10

**Authors:** Priscila Silva Figueiredo, Taynara Nogueira Martins, Luciana Marçal Ravaglia, Glaucia Braz Alcantara, Rita de Cássia Avellaneda Guimarães, Karine de Cássia Freitas, Ângela Alves Nunes, Lincoln Carlos Silva de Oliveira, Mário Rodrigues Cortês, Flavio Santana Michels, Mônica Cristina Toffoli Kadri, Iluska Senna Bonfá, Wander Fernando de Oliveira Filiú, Marcel Arakaki Asato, Bernardo Bacelar de Faria, Valter Aragão do Nascimento, Priscila Aiko Hiane

**Affiliations:** 1 Post-Graduate Program in Health and Development in the Mid-West Region, Federal University of Mato Grosso do Sul, Campo Grande 79070-900, MS, Brazil; priscilas.figueiredo@hotmail.com (P.S.F.); kcfreitas@gmail.com (K.d.C.F.); aragao60@hotmail.com (V.A.d.N.); priscila.hiane@ufms.br (P.A.H.); 2 Chemistry Institute, Federal University of Mato Grosso do Sul, Campo Grande 79070-900, MS, Brazil; taynaranogueiramartins@hotmail.com (T.N.M.); luciana.ravaglia@ufms.br (L.M.R.); glaucia.alcantara@ufms.br (G.B.A.); lincoln.oliveira@ufms.br (L.C.S.d.O.); mariocortess40@gmail.com (M.R.C.); 3 Program in Biotechnology, Universidade Católica Dom Bosco, Campo Grande 79117-900, MS, Brazil; nunysnutri@yahoo.com.br; 4 Optics and Photonics Group, Institute of Physics, Federal University of Mato Grosso do Sul, Campo Grande 79070-900, MS, Brazil; santanamichels@gmail.com; 5 Faculty of Pharmaceutical Sciences, Food and Nutrition, Federal University of Mato Grosso do Sul, Campo Grande 79070-900, MS, Brazil; monica.kadri@ufms.br (M.C.T.K.); iluskasenna@hotmail.com (I.S.B.); wander.filiu@gmail.com (W.F.d.O.F.); 6 Medical School, Federal University of Mato Grosso do Sul, Campo Grande 79070-900, MS, Brazil; marcel_arakakiasato@hotmail.com (M.A.A.); bacelarfaria@gmail.com (B.B.d.F.)

**Keywords:** vegetable oils, metabolomic assay, thermal stability, oil quality, leukocyte migration

## Abstract

Different fatty acid proportions produce potential inflammatory and metabolic changes in organisms. However, the evidence for how each fatty acid mediates the metabolic pathway, and its lipid stability remains controversial. To resolve this controversy, the present study investigated the metabolic effects of cold-pressed linseed (LG), coconut (CG), and baru (BG) oils in comparison to those of soybean oil (SG) in mice, in terms of their oil characterization and stability. The quality analysis showed less oxidative behavior among PUFA-rich oils (SO, BO, and LO, with induction periods lower than 2 h compared to 39.8 h for CG), besides the high contents of tocopherols and carotenoids in SG and LG. In the experimental study, CG presented higher triglyceride (257.93 ± 72.30) and VLDL-cholesterol levels (51.59 ± 14.46, *p* < 0.05), while LG reduced LDL levels (59.29 ± 7.56, *p* < 0.05) when compared to SG (183.14 ± 22.06, 36.63 ± 4.41 and 131.63 ± 29.0, respectively). For visceral fats, the adiposity index was lower for BG (7.32 ± 3.13) and CG (9.58 ± 1.02, *p* < 0.05) in relation to SG (12.53 ± 2.80), and for leukocyte recruitment, CG presented lower polymorphonuclear (PMN) (*p* < 0.0001) and mononuclear (MN) (*p* < 0.05) cell infiltration, demonstrating anti-inflammatory potential. In NMR-based metabolomics, although CG presented higher values for the glucose, lactate, and LDL/VLDL ratio, this group also evidenced high levels of choline, a lipotropic metabolite. Our study emphasized the controversies of saturated fatty acids, which impair serum lipids, while alfa-linolenic acid presented cardioprotective effects. However, coconut oil also has a positive immunomodulatory pathway and was found to reduce visceral bodyfat in mice. Therefore, for future applications, we suggest a combination of lauric and al-fa-linolenic acid sources, which are present in coconut and linseed oil, respectively. This combination could be less obesogenic and inflammatory and exert cardioprotective action.

## 1. Introduction

Recently, using randomized controlled trials, the UK Scientific Advisory Committee on Nutrition (SACN) demonstrated the positive impact of reducing saturated fatty acid (SFA) intake to improve cardiovascular health and glycemic control after replacing SFA with polyunsaturated fatty acid (PUFA). Since these metabolic issues are usually related to excess adipose tissue and its endocrine activity, it is worthwhile to further evaluate and determine the impacts of lipid consumption on the functions of this organ [1].

Several years ago, a study was performed on overweight/obese humans, which demonstrated a correlation between different fatty acids and the size, number, and localization of adipocytes. A positive association was found with polyunsaturated fatty acid (PUFA) that reduced adipocyte size, while dietary saturated fatty acid (SFA) was related to an increase in fat-cell number and size [2].

The importance of measuring abdominal fat is associated with the correlation between this type of fat and a greater risk of developing metabolic diseases, such as diabetes and cardiovascular events. Abdominal fat is composed of subcutaneous adipose tissue (SCAT) and visceral adipose tissue (VAT). The latter is related to greater metabolic damages since it has more cells, innervations, and vases as a greater percentage of major adipocytes, in addition to its lower preadipocyte differentiating capacity and a larger number of inflammatory and immune cells, creating a predisposition for chronic inflammatory conditions [3].

Considering an anti-inflammatory approach, it is suggested to increase the consumption of unsaturated fatty acids, especially n-3 PUFA [4], whose main sources are marine fish and their derivates, such as oil fish, which contain eicosapentaenoic acid (EPA) and docosahexaenoic acid (DHA), as well as alpha-linolenic acid (ALA), which could give rise to EPA and DHA endogenously throughout an enzymatic cascade [5].Like with the other n-3 PUFAs mentioned, ALA is considered an essential fatty acid and, therefore, needs to be obtained through food, its main sources being vegetables, such as green leafy vegetables and certain nuts (e.g., walnuts, rapeseed, and linseed) [1]. 

Other unsaturated fatty acid sources include soybean oil, which is part of the group of oils most commonly produced and consumed worldwide [6]; a source of n-6 PUFA known as linoleic acid (LA); and baru oil, derived from baru (DipteryxalataVog), a fruit of the Brazilian Cerrado, which has high oleic acid content—the primary example of a monounsaturated fatty acid (MUFA) [7], which is very close to the oleic acid content of olive oil [8] and associated with cardioprotective effects [9]. On the other hand, as the main plant source of saturated fatty acids, there is coconut oil, whose consumption has increased considerably in recent years [6], primarily due to its nutritional properties related to general health improvements [10], contrary to the recommendation of reducing SFA.

In addition to assessing the different metabolic impacts of these lipid sources, it is also worth highlighting the importance of better understanding their analytical properties and determining their quality and stability. Well-validated and recommended techniques are presently used to study these properties, such as quality and identity indexes and optical and thermal stability techniques. In addition, for the widely consumed lipid sources mentioned above, further studies are needed to develop a clinical and analytical correlation, as a way to expand the existing evidence for future recommendations to the population.

Soybean, linseed, and coconut oil are commonly used in food, while baru oil has grown in popularity in recent years due to its antioxidant properties. Additionally, these oils are important food sources of unsaturated fatty acids, with different proportions of n-6 PUFA, n-3 PUFA, MUFA, and SFA. Metabolomics remain under-explored as a bioanalytical technique for researching the effects of consuming different types of fatty acids. Moreover, the effects of these fatty acids have rarely been studied through metabolomic analysis. Therefore, the present study aimed to characterize cold-pressed linseed, coconut, and baru oil in comparison to soybean oil, according to their nutritional quality, leukocyte infiltration, and NMR-based metabolomic effects, and analytical approaches, in mice.

## 2. Materials and Methods

### 2.1. Vegetable Oils

#### 2.1.1. Raw Material

Cold-pressed golden linseed, coconut, and soybean oils were purchased from Pazze™ Food Industry (Panambi, Rio Grande do Sul, Brazil), while cold-pressed organic baru oil was obtained from Ybá Pure Oils Industry and Commerce (Alto Paraíso de Goiás, Goiás, Brazil). The oils were characterized and also used in the preparation of the experimental diets.

##### Fatty Acid Profiles and Indices of Nutritional Quality

Fatty acid methyl esters were analyzed by gas chromatography (GC-MS 2010, Shimadzu, Japan) to obtain their individual peaks. The equipment used a flame ionization detector (FID) and a capillary column (BPX-70, internal diameter of 0.25 mm, 30 m long, and 0.25 mm thick film). The injector and detector temperatures were 240 °C. The initial column temperature was maintained at 70 °C for 2 min and then increased to 10 °C/min until reaching 150 °C, followed by an increase to 240 °C at 5 °C/min for 5 min. Individual FAME peaks were identified by comparing their relative retention times with the FAME standard (fatty acid methyl ester) (Supelco C22, 99% pure).

Based on the composition of free FAs, we assessed the nutritional quality using three different indices: the atherogenic index (AI) (Equation (1)), the thrombogenic index (TI) (Equation (2)), and the hypocholesterolemic: hypercholesterolemic (HH) ratio (Equation (3)) [11]:

(1) $$\mathrm{AI}=\frac{C_{12:0}+4\times C_{14:0}+{\;C}_{16:0}}{\sum^{\;}\mathrm{MUFA}\;+\sum^{\;}\omega 6+\sum^{\;}\omega 3}$$

(2) $$\mathrm{TI}=\frac{C_{14:0}+{\;C}_{16:0}+{\;C}_{18:0}}{0.5\times \sum^{\;}\mathrm{MUFA}+0.5\times \sum^{\;}\omega 6+3\times \sum^{\;}\omega 3}$$

(3) $$\mathrm{HH}=\frac{C_{18:1\mathrm{cis}9}+{\;C}_{18:2\omega 6}+{\;C}_{20:4\omega 6}+{\;C}_{18:3\omega 3}+{\;C}_{20:5\omega 3}+{\;C}_{22:5\omega 3}+{\;C}_{22:6\omega 3}}{C_{14:0}+{\;C}_{16:0}}$$

##### Carotenoid Levels

To quantify total carotenoids, the sample was solubilized in petroleum ether, and the absorbance was detected at 450 nm using a spectrophotometer [12].

##### Quality and Identity Analyses of Oils

The refractive index, peroxide value, iodine value, acidity index, and saponification index were evaluated to characterize the quality and identity of the linseed, coconut, soybean, and baru oils. All analyses were performed in triplicate in accordance with the methods proposed by the Association of Official Analytical Chemists [13].

##### Optical Characterization 

For molecular absorption analysis, the vegetable oils were prepared at two concentrations, 0.13 and 50.0% (*v*/*v*), by diluting the oils in hexane (Vetec™ > 99%, spectroscopic grade). Absorption spectra were collected in the 200 to 800 nm range using a UV–Vis spectrometer LAMBDA 265 UV−Vis (PerkinElmer™). Fluorescence excitation–emission matrices were measured in excitation and emission ranges of 275 to 475 nm and 290 to 700 nm, respectively. The fluorescence data were collected without any sample preparation (i.e., undiluted oils) using a bench spectrofluorimeter FS-2 (Scinco™). All measurements were performed in triplicate at room-temperature.

##### Oxidative Stability 

Oxidative stability was determined according to the European norm EN 14,112 with the aid of an 893 Professional Biodiesel Rancimat (Metrohm™) device. During the analysis, 3.0 g of the vegetable oils was subjected to constant heating at 110 °C in the presence of airflow of 10 L.h^−1^. The induction period (IP) of the vegetable oils was determined by analyzing the conductivity increase caused by volatile products generated during oil oxidation. 

##### Thermogravimetric Analysis (TGA)

Our vegetable oils were evaluated for their thermal stability in a TGA–Q50 thermogravimetric analyzer (TA Instruments, New Castle, DE, USA). Approximately 5 mg of each sample was used, with platinum crucibles applied for support. The heating rate was set to 10 °C min^−1^, from room-temperature to 900 °C, in a synthetic air atmosphere with a purge flow of 60 mL min^−1^ in the oven. To ensure the accuracy of the results, the TGA-Q50 device was calibrated for mass thermobalance using standard weights of 100 and 1000 mg before obtaining the TG-DTG curves. Temperature was adjusted by determining the Curie point of nickel (358 °C).

#### 2.1.2. In Vivo Analyses 

##### Animal and Experimental Design

Animal protocols followed the relevant ethical rules and guidelines, and the experimental protocol was approved by the Ethics Committee for Animal Use (Protocol n^o^. 868/2017), which is essential in the International Guiding Principles for Biomedical Research Involving Animals (CIOMS), Genebra, 1985; the Universal Declaration of Animal Rights (UNESCO), Bruxelles, Belgium, 1978; and the guidelines of the National Health Institutes on the use and care of laboratory animals. 

We used 116 Swiss mice (*Mus musculus*), which were randomly assigned to three experimental groups and a control group (CG for the coconut oil group; LG for linseed oil; BG for baru) and a control group with soy oil (SG); all received diet formulations according to the American Institute of Nutrition (AIN-93M) [14], varying only in the lipid source included in the diet, which consists of 7% total lipids over 60 days of treatment. The groups were maintained under a 12/12 h light/dark cycle at 22 °C. The animals were weighed weekly on a semi-analytic balance (Marte-Modelo™ AS 5.500, São Paulo, Brazil) and received food and water *ad libitum*. 

##### Morphometric and Histological Parameters

Food consumption was weighed weekly (grams/day). At the end of the experiment, after nocturnal fasting, we recorded the body weights. Next, we anesthetized the mice with isoflurane for blood sampling through the inferior cava vein, and then the animals were euthanized by bleeding. The visceral fat sites (epididymal, mesenteric, retroperitoneal, perirenal, and omental) and liver were collected and weighted, and a portion of the liver was stored in formalin for later histological analysis.

We also calculated the adiposity index (AI) according to the following formula: sum of visceral fat sites/final body weight × 100 [15].

##### Serum Analyses 

Blood samples were centrifuged at 3000 rpm for 5 min, and the serum was separated and stored for further analysis (Fanem™, Excelsa II, 206 BL, São Paulo, Brazil) to determine the biochemical parameters of the lipid profiles and glycaemia using the enzymatic–colorimetric method and spectrophotometric readings [16,17,18].

##### NMR-Based Metabolomic Analysis

For NMR-based metabolomics, 120 μL of the serum sample was added to 450 μL of deuterated phosphate buffer (pH 7.2) containing DSS-d_6_ (hexadeuterated sodium 2,2-dimethyl-2-silapentane-5-sulfonate) as an internal standard, centrifuged at 12,000 rpm for 3 min. Subsequently, 550 μL of the remaining solution was transferred to a 5 mm NMR tube.

The ^1^H NMR spectra were obtained using an AVANCE NEO 500 spectrometer (11.75T) operating at 500 MHz for the ^1^H frequency, with NOESYGPPR1D and CPMGPR1D as the pulse sequences. The NOESYGPPR1D parameters were as follows: an acquisition time (AQ) of 2.2282 s, a spectral window (SW) of 29.4040 ppm, 128 scans (NS), receiver gain (RG) of 101, a number of time-domain points (TD) of 64 k, and a relaxation time (d1) of 6 s. The CPMG pulse sequence was as follows: D−[−90−(τ−180−τ)n−FID], in which D = 6.0 s was chosen to allow T1 relaxation; τ = 0.300 ms was fixed after optimization to permit the attenuation of broad signals (“T2 filter”) and the refocusing of spin-coupled multiplets; *n* = a fixed loop of 60 cycles; and water suppression was included in the CPMG sequence. The magnetic field homogeneity was automatically adjusted, and its quality was checked with comparative values using the half height of the internal standard signal for all samples.

^1^H NMR processing was performed via manual adjustment of the phase and baseline, followed by automatic calibration using DSS-d_6_ as the internal standard reference at 0.00 ppm. To improve the signal-to-noise ratio, line broadening (LB) of 0.3 Hz and zero-filling processing (SI = TD) were used, respectively.

NMR metabolite assignments were conducted using CPMG spectra due to reduction in the broad signals from macromolecules and two-dimensional NMR spectra (^1^H-^1^H COSY, ^1^H-^1^H TOCSY, ^1^H-^13^C HSQC, ^1^H-^13^C HMBC, and Jres). The metabolite assignments were confirmed using the HMDB database, the spectra of standard substances, and the literature [19,20,21]. The metabolite assignments are shown in Table S4.

The relative proportion determination was performed by integrating the non-overlapping signals of some metabolites highlighted in the multivariate analyses, using the DSS-d_6_ signal as the internal standard reference.

##### Leukocyte Recruitment Induced by Carrageenan (Cg)

This test was conducted according to the method described by Souza and Ferreira [19]. After pre-treatment, the animals received 0.5 mL of 1% Cg i.p. Four hours after Cg injection, the animals were euthanized with isoflurane inhalation and subsequent exsanguination through the portal vein, and leukocyte influx to the peritoneal cavity was then evaluated. The peritoneal cavity was washed with 3 mL of 0.1% heparinized buffered saline (PBS) containing 1% bovine serum albumin (3%). Afterwards, peritoneal exudate was collected, and total and differential cell counts were performed. For the total leukocyte count, 20 μL of the exudate was removed, and 380 μL of Turk’s liquid was added, with quantification performed in a Neubauer chamber. For the differential count, the exudate was centrifuged at 700 rpm/5 min. The supernatant was discarded, and the pellet was resuspended in 200 μL of 3% bovine albumin and distributed on slides stained with HEMA3^®^ dye. Next, the differential cell count was determined. The cells were classified as mononuclear or polymorphonuclear according to morphological criteria. The results were expressed as the number of cells per mm^3^.

### 2.2. Statistical Analyses

Data are expressed as the mean ± SD. Data were subjected to parametric analysis by one-way analysis of variance followed by Bonferroni’s test as a post hoc procedure. Tukey’s test was utilized to determine the identity indexes of the oils. All evaluations were considered significant at 95% (*p* < 0.05). Histological data, on the other hand, were presented descriptively, with absolute (n) and relative (%) frequencies represented in a table. A chi-squared test was used to assess the association in histological analysis, followed by Bonferroni correction, using the statistical program Bioestat 5.0. The level of significance adopted was *p* < 0.05. In the absence of observing any analyzed variable, these values were disregarded in the statistical analysis.

The processed ^1^H NMR spectra were transferred to the AMIX™ (Bruker) software for bucketing using a simple rectangular integration mode with a 0.02 ppm bucket width and scaled by the sum of the total intensity. After bucketing, the MetaboAnalyst™ software was used for chemometric treatment. Unsupervised and supervised pattern recognition algorithms were employed using Pareto preprocessing: Principal Component Analysis (PCA), Partial Least Squares–Discriminant Analysis (PLS-DA), and Sparse Partial Least Squares–Discriminant Analysis (sPLS-DA).

For NMR, we used the relative proportion of metabolites previously highlighted from multivariate analysis, and the normality test was evaluated using the Shapiro–Wilk method. According to the test results, ANOVA (for parametric data) and Mann–Whitney/Kruskal–Wallis were applied to parametric (*p* > 0.05) and non-parametric (*p* < 0.05) data, respectively. The statistical results are shown in a box-plot.

## 3. Results 

### 3.1. Fatty Acid Profiles and Nutritional Quality of Oils 

Supplementary Table S1 presents the fatty acid profile and nutritional quality of each oil. Soybean oil (SO) represents a linoleic acid (LA) source; baru oil (BO) represents a MUFA source, especially for oleic acid (similar to olive oil, which has between 55 and 88% MUFA) [8]; coconut represents a vegetable source of saturated fatty acid (SFA); and linseed oil (LO) represents a very good source of ALA, an n-3 PUFA type. LO was also the greatest PUFA source, with PUFA representing close to 65% of LO’s total fatty acids.

Obtaining the fatty acid profiles through chromatography allowed us to evaluate the nutritional quality of oils according to equations of nutritional indexes. Here, AI and TI demonstrated the ability of FAs to favor or prevent atherosclerosis and coronary thrombosis, considering their effects on serum cholesterol and low-density lipoprotein (LDL) concentrations. In this study, both AI and TI were lower in oils predominant in unsaturated acids, SO, BO, and LO and higher in CO. 

### 3.2. Analytical Oils Approach 

#### 3.2.1. Quality and Oils Stabilities

Although linseed oil presented higher carotenoid levels, its acidity index was also the highest (Table S2), indicating vulnerability to oxidation compared to the other oils. Another quality parameter is the peroxide index, which indicates the oxidative rancidity process in foods, thereby demonstrating lipid stability, since the accumulation of peroxide—such as aldehydes, ketones, and conjugated dienes—results in an unstable and decomposed status [20]. Baru oil presented the highest peroxide value compared to the control oil; nonetheless, this value and acidity level are in accordance with the virgin olive oil recommendation, which were less than 1.5% and 20 mEq/O_2_ for acidity and peroxide, respectively [21,22], since there is no specific regulation for this oil, and its composition is very similar to that of olive oil.

The Rancimat test demonstrated faster degradation for linseed oil (Figure S1). As shown in the results, the linseed, baru, and soybean oils presented low oxidative stability, with induction period values of 0.9, 1.0, and 1.7 h, respectively. Coconut oil had a high induction period of 39.8 h. This high oxidative stability could be associated with coconut oil’s fatty acid composition (Table S1) and natural antioxidant content. 

This result indicates that the high thermal stability of CO is closely related to its high content of saturated fatty acids (Table S1). Notably, CO presented more than 81% saturated fatty acids, while LO, BO, and SO contained about 87, 80, and 72% unsaturated fatty acids, respectively.

#### 3.2.2. Optical Characterization

Figure S2 shows that all vegetable oils presented strong absorption below 350 nm, which was associated with the content of tocopherols and fatty acid methyl esters present in the oils, such as alpha tocopherol, methyl linoleate, and stearate [23,24], which indicate unsaturated fatty acids, especially linoleic and oleic acid. Additionally, absorption bands in the 400 to 500 nm range were observed only for the linseed and soybean oils (inset of Figure S2), indicating that both oils presented higher contents of carotenoids, as these absorption bands are characteristics of carotenoid molecules [23]. This observation is in accordance with the results described in Table S2 through the analysis of carotenoids.

Figure S3 illustrates the presence of different fluorophores in the vegetable oils. In accordance with the absorption results, carotenoid emission bands in the 500 and 600 nm range were observed for the linseed and soybean oils (Figure S3a,b). The fluorescence results additionally revealed the presence of chlorophyll molecules in the linseed and soybean oils as illustrated by the typical chlorophyll fluorescence emission at around 670 nm (Figure S3a,b) [24,25]. 

Furthermore, it is important to highlight that the emissions of tocopherols (300 to 400 nm range) were better visualized for the coconut oil (Figure S3d). This observation was likely due to the coconut oil’s lower capacity of absorption below 325 nm, which avoided the inner-filter effect caused by the reabsorption process when fluorescence measurements were performed on the undiluted oils [24].

#### 3.2.3. Thermogravimetric Analyses

The TG curves in Figure S4 indicate that most of the evaluated vegetable, baru, linseed, and soybean oils experienced a mass loss around 200 °C. These losses were mild and represented around 2% of the weight of the samples, which was justified by the loss of moisture. The second stage of degradation was observed more significantly at around 250 °C and was more strongly associated with the presence of unsaturated fatty acids, such as oleic, linolenic, and linoleic, which were present in large quantities in baru, flaxseed, and soybean, respectively, in addition to presenting a loss greater than 95% at between 450 and 470 °C. The expressive degradation temperature of coconut is around 350 °C, at which point we observed a mass loss of 80.0%. This loss was related to the oxidative decomposition of saturated fatty acids, which feature the lowest number of carbon atoms (6 at 16 carbons) [26,27]. 

### 3.3. Experimental Study

#### 3.3.1. Morphometric Parameters

Based on the in vivo study (Table 1), the body weight, food ingestion, and liver weight were different between experimental groups. As a method for measuring the visceral fat tissue in experimental models, intra-abdominal fat is weighed after dissection, which provides a functional and accessible way to estimate this variable [28].

Considering the visceral fats, animals that consumed baru oil showed improvements in epididymal, mesenteric, and retroperitoneal sites of body fat. These areas presented the largest amounts of visceral fat and agreed with the adiposity index, which was also reduced in this group (Table 1). Interestingly, the CG group showed positive results in the mesenteric fat and adiposity index (*p* < 0.0001), despite being a source of saturated fatty acids. However, the control group (soybean oil) did not show any significant results for body composition.

#### 3.3.2. Effects of Oils on Liver Tissue

The prevalence of microvesicular steatosis was significantly higher in SG compared to the other groups (*p* = 0.03). However, the other groups did not differ from each other. Steatosis in zone 3 was observed only in the five analyses of the control group and was analyzed together with zone 1 due to the small sample size for each classification. However, the location of steatosis was not associated with the treatment group (*p* = 0.59). No group featured nuclear glycogenation and, therefore, there was no statistical analysis of this variable.

Although there was no significant association between the other treatments for apoptosis (*p* = 0.68), ballooning (*p* = 0.06), steatosis (*p* = 0.94), Mallory’s hyaline (*p* = 0.34), steatosis location (*p* = 0.05), or lobular inflammation (*p* = 0.05), the latter variable tended to be less prevalent in LG and presented an increased value for the control group. The values are detailed in Table S3.

When analyzing the slide images (Figure S5), we noted the presence of macrogoticular steatosis, especially in acinar zone 3 of the CG group, as well as macrogoticular steatosis, microgoticular steatosis, and accentuated ballooning in acinar zone 3 of the SG group. Slides BG and LG presented preserved liver parenchyma.

#### 3.3.3. Biochemical Profiles 

Regarding serum parameters, LG had a greater reduction (*p* < 0.05) in LDL Cholesterol when compared to the control (Table 2), and lipoprotein was associated with an accelerated atherosclerosis process [29]. LG also presented better high-density lipoprotein (HDL) levels (*p* < 0.05), which is a lipoprotein that offers anti-atherogenic effects by reversing cholesterol transport from the peripheral tissues to the liver [30]. Additionally, triglyceride and VLDL-cholesterol (a triacylglycerol-rich particle) levels were higher in the CG group (*p* < 0.05). 

#### 3.3.4. NMR-Based Metabolomics of the Serum Samples

**Figure 1:**
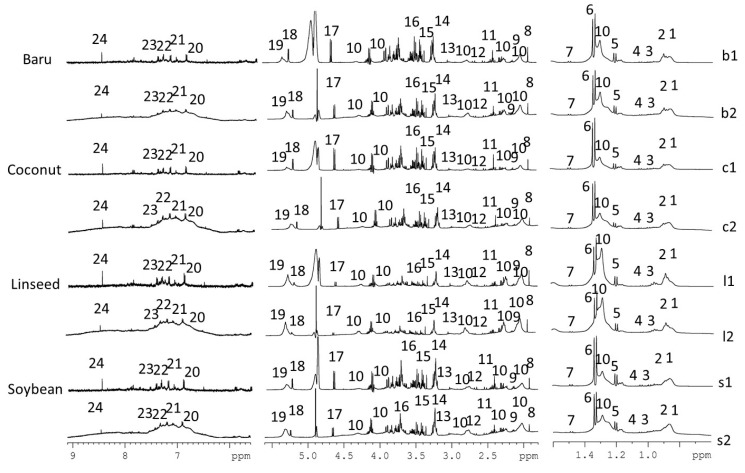
Representative ^1^H NMR spectra (500 MHz, D_2_O buffer pH 7.2) of mice serum from CPMGPR1D (b1, c1, ll, s1) and NOESYGPPR1D (b2, c2, l2, s2) pulse sequences containing the assigned metabolites: 1, 2—LDL/VLDL, 3—leucine, 4—valine, 5—β-hydroxybutyrate, 6—lactate, 7—alanine, 8—acetate, 9—glutamine/glutamate, 10—lipids, 11—succinate, 12—citrate, 13—creatine/creatinine, 14—choline, 15—phosphocholine, 16—taurine, 17—β-glucose, 18—α-glucose, 19—unsaturated fatty acids, 20—tyrosine, 21—histidine, 22—L-phenylalanine, 23—tryptophan, and 24—formate.

Representative ^1^H NMR spectra (Figure 1) revealed the 24 assigned metabolites from 1D and 2D NMR data: alanine, β-hydroxybutyrate, lactate, α/β-glucose, citrate, acetate, creatine/creatinine, LDL/VLDL, fatty acids, unsaturated fatty acids, leucine, valine, glutamine/glutamate, succinate, choline, phosphocholine, taurine, tyrosine, histidine, 1-phenylalanine, tryptophan, and formic acid. Details about NMR assignments are provided in Table S4.

Considering the complexity of the ^1^H NMR data, the matrix complexity indicated the need for a more refined treatment to compare the differences and similarities between the sample groups. Thus, chemometric analyses were applied to compare differences and similarities among groups and extract the maximum chemical and metabolic information about the studied serum samples.

**Figure 2:**
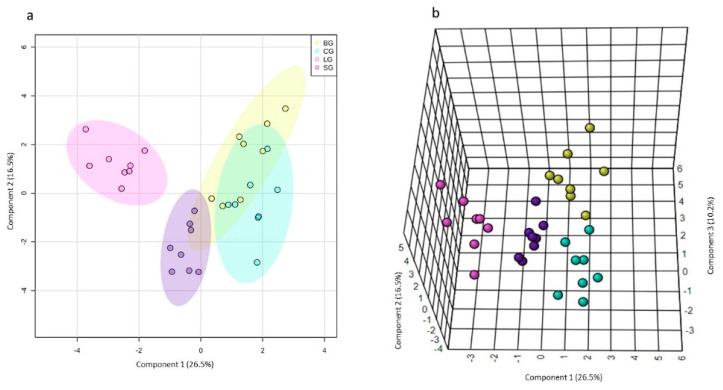

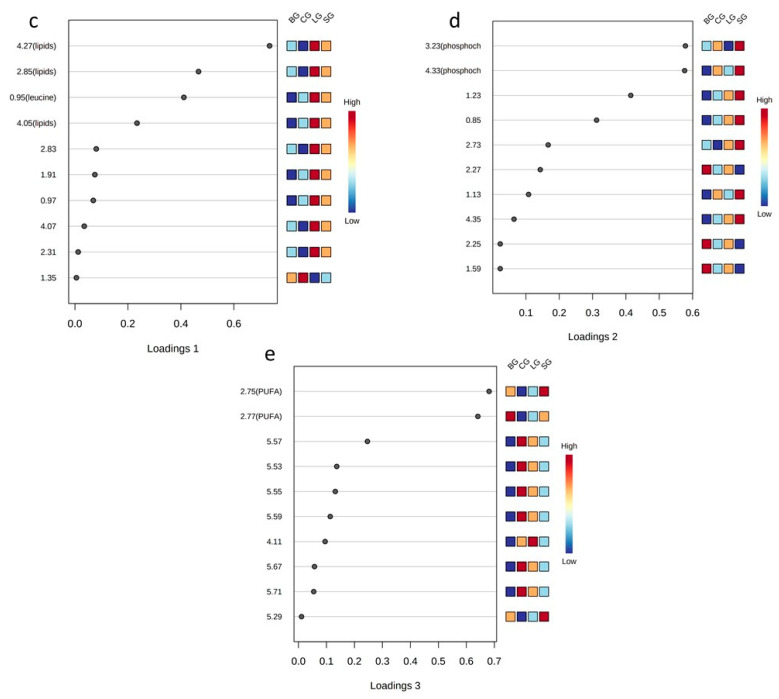
The 2D (**a**) and 2D (**b**) sPLS-DA score plots and loadings for components 2 (**c**–**e**) obtained from the ^1^H NMR spectra (NOESYGPPR1D) of the serum samples from Swiss mice fed diets based on baru, coconut, linseed, and soybean oils. Legend: baru (BG), coconut (CG), linseed (LG), and soybean oil (SG) groups.

PCA, an unsupervised pattern recognition method, was not sufficient to distinguish the four sample groups, especially due to the baru group’s dispersion (Figure S6a). On the other hand, when the baru group was removed, the PCA for the remaining samples revealed differences between the linseed group and coconut and soybean groups (Figure S6c). Both PCAs presented α/β-glucose, LDL/VLDL, lactate, and fatty acid metabolites as loadings (Figure S6b,d). In order to maximize the differences between the studied groups, we used sPLS-DA as a supervised pattern recognition method (Figure 2a,b). The sPLS-DA algorithm was used to select variables considered important to explain the separation between the different declared groups and the similarities between similar groups [31].

As seen in Figure 2a,b, only the linseed group had well-defined separation from others in the 2D sPLS-DA score plot. (i.e., the baru group and coconut and soybean group). However, in the 3D sPLS-DA score plot, we observed that the four studied groups were allocated differently and that the primary separation occurred on component 1. Component 2 influenced the linseed group’s separation from the coconut and soybean groups, and. finally, component 3 was considered essential to separate the baru group from the coconut and soybean groups. For the sPLS-DA loading plots, each component (1, 2, and 3) demonstrated which variables were selected by the algorithm to justify the separation between groups in each component (Figure 2c–e). 

**Figure 3:**
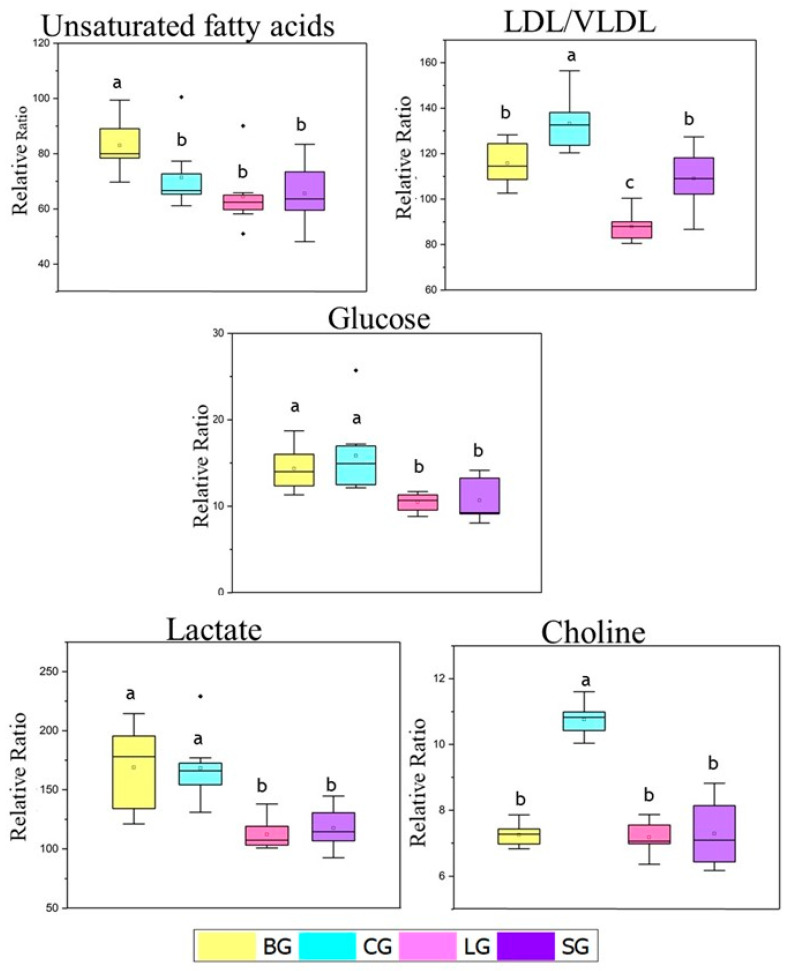
Relative proportion of unsaturated fatty acid, LDL/VLDL, glucose, choline, and lactate metabolites identified from ^1^H NMR spectra in the serum of mice fed with baru, coconut, linseed, and soybean oils. The relative ratio was obtained by integrating the relevant signals with reference to the DSS-d-6 signal Differences within unsaturated fatty acids and glucose levels were tested with Krus-kal–Wallis and Mann–Whitney tests. Differences within LDL/VLDL, choline and lactate levels were tested with ANOVA tests. The same letters represent no significative differences. Legend: baru (BG), coconut (CG), linseed (LG), and soybean oil (SG) groups.

Thus, in the sPLS-DA loading 1 plot (Figure 2c), the important variables are 4.27, 4.05, 2.85, and 0.95 ppm, among which the buckets for 4.27, 4.05, and 2.85 ppm correspond to lipids, while the signal at 0.95 ppm corresponds to leucine. These signals presented higher levels in the linseed group, followed by the soybean, baru, and coconut groups, indicating that diets based on unsaturated oils are responsible for high levels of lipids and leucine in the serum samples. The increase in lipids in Swiss mice that ingested unsaturated oils is illustrated in Figure 3. 

In the sPLS-DA loading 2 plot (Figure 2d), the most important variables are 3.23 and 4.33 ppm, corresponding to phosphocholine. Figure 2d also illustrates high levels of phosphocholine in the soybean group. This metabolite is an intermediary in the formation of phosphatidylcholine, an important phospholipid involved in cell secretion processes, such as those of lipoproteins [32]. 

In the sPLS-DA loading 3 plot (Figure 2e), the variables at 2.77 and 2.75 ppm, which correspond to the bis-allylic hydrogen signals present in polyunsaturated fatty acid (PUFA), revealed the separation of the baru group from the others, especially from the coconut group. This result is in accordance with coconut oil’s composition, which is a source of saturated fatty acids (Table S1). 

Subsequently, to determine how metabolic differentiation occurs between the studied groups, univariate statistical analyses were performed using the relative proportion NMR data for certain metabolites highlighted in the multivariate analyses: unsaturated fatty acids, LDL/VLDL, glucose, lactate, and choline (Figure 3).

**Figure 4:**
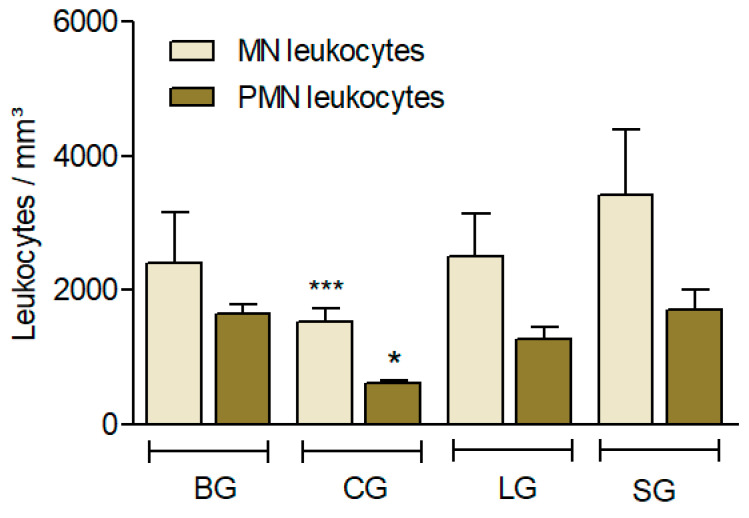
Effect of different diets (oils) on the leucocyte influx induced by Carrageenan (Cg). The animals received diet formulations of AIN for 60 days, with only the lipid sources modified (SG: soy oil, control; BG: baru oil; LG: linseed oil; CG: coconut oil). Four hours after the i.p. administration of Cg, the influx of (MN) and polymorphonuclear leukocytes (PMN) was evaluated. Results are expressed as the mean ± SD (*n* = 6). ANOVA followed by a Bonferroni test. * *p* < 0.05; *** *p* < 0.001 (compared to the control, SG).

For the relative proportions of LDL/VLDL, we observed the highest values for the coconut group, intermediate values for the baru and soybean groups, and the lowest levels for the linseed group (Figure 4). These results are in agreement with the biochemical parameters (Table 2) and nutritional quality indexes (atherogenic and thrombogenic (Table S1)), which were also elevated for CG, as represented by Swiss mice fed a diet rich in oils mainly consisting of saturated fatty acids (coconut group). As mentioned above, CG also increased triacylglycerol levels, which is a predominant component in the molecules of VLDL. 

Higher levels of glucose were observed in the baru and coconut groups in the relative proportion data, in contrast to the values in the linseed and soybean groups (Figure 3). 

Finally, choline was selected to perform the NMR relative proportion test since choline is a precursor of phosphocholine, an important metabolite in sPLS-DA loading 2 (Figure 2d). As shown in Figure 4, the coconut group presented higher values of choline compared to the other groups; the coconut group was the only group with a significant difference from the others.

#### 3.3.5. Leukocyte Recruitment Induced by Carrageenan

Leukocyte recruitment used an experimental model to evaluate the anti-inflammatory effects, which consisted of quantifying the leukocyte cells in the peritoneal cavity of the mice.

In this study, CG showed less leukocyte recruitment, suggesting a greater anti-inflammatory response (*p* < 0.001) in PMN cells and MN cells (*p* < 0.05) (Figure 4).

## 4. Discussion

Our study evaluated the ingestion of four different lipid sources in rodents and their metabolic and metabolomic effects after 60 days. Considering the nutritional quality indexes, the groups with the highest contents of unsaturated fatty acids, especially PUFA (LO, BO, and SO groups), presented the highest hypocholesterolemic indexes, which are associated with positive anti-inflammatory effects for neurodevelopment, especially those related to cardiovascular health [33]. 

Following the main recommendations in recent years regarding cardiovascular health, our results are in agreement with the proposal that PUFA sources are cardioprotective since only the linseed group (LG) demonstrated better values for serum lipids (higher for HDL and lower for LDL and triglycerides). As mentioned above, linseed oil is a source of α-Linolenic acid (ALA), an n-3 PUFA, and has been associated with promising results, such as anti-cancer, neuroprotective, and anti-osteroporotic properties, as well as protection against cardiovascular damage, minor inflammation, and cellular lipotoxicity [5].

Although coconut oil, as shown in Table S1, had the greatest content of SFA, especially medium-chain triglyceride (MTC), C10 and C12 fatty acids [34], as also presented the highest saturated fatty acids in NMR assay (Figure 4), which should be included in the recommendations for dietary restrictions. However, some paradoxes emerged in our study since the CG group featured impaired serum levels in the metabolomic assay, especially the levels of LDL, triglycerides, and VLDL-cholesterol (Table 2), as well as glucose (Figure 3). On the other hand, this group featured lower visceral fat (Table 1) content, as did the BG group.

The resulting glucose levels agree with the lactate values observed for both CG and BG (Figure 3), as this nutrient is a metabolite formed in the glycolytic pathway and an immediate source for pyruvate formation, which is sent to the mitochondria for complete oxidative combustion. Another important point is the presence of large amounts of medium-chain triglycerides in the CG, whose absorption is faster as an energy source. These triglycerides would also explain the lower adiposity index found in this group (Table 1). This is similar to the model in another study [35], which demonstrated a reduction in body fat with coconut oil when compared to the soybean oil group, as well as metabolic diseases such diabetes, insulin resistance, and cardiovascular problems, which are associated with visceral adipose tissue (VAT) [3].

Although BG is a source of oleic acid, a meta-analysis [36] showed that this fatty acid presents benefits only when in a Mediterranean food context, in which the proportions of fat (with a notable reduction in saturated and linoleic acids) are well balanced, and the amount of antioxidants is increased. As demonstrated before, the baru oil used in this study contained few antioxidant compounds, such as tocopherols and carotenoids (as shown in Table S2 and Figures S2 and S3), which could impact the metabolomic results.

Additionally, CG also presented higher levels of choline, a metabolite considered an essential nutrient responsible for the biosynthesis of phospholipids (components of cell membranes), such as phosphatidylcholine, which participates in the release of VLDL-type lipoproteins (which has high triacylglycerol content) from the liver. In addition, choline is considered a lipotropic substance since it prevents lipid accumulation in the liver [37]. Choline also participates in the production of the acetylcholine neurotransmitter, thereby helping to reduce homocysteine, which is associated with cardiac damage [38,39]. 

In relation to the leukocyte infiltration model, Carrageenan (Cg) was injected into the peritoneal cavity and induced leukocyte influx, as well as accumulation of exudate at the inflammatory site [40,41]. This influx during acute inflammation was characterized by the presence of leukocytes, mainly polymorphonuclear (PMN) cells [42]. Considering the complexity of multicellular organisms, leucocytes generally move long distances and initiate the site of infection or inflammation after encountering multiple microenvironments and various chemo-attractants. This migration progress, known as chemotaxis, plays a fundamental role in inflammation, angiogenesis, wound healing, and many other complex physiological processes [43]. The CG group also presented lower recruitment than the other groups (Figure 4), which could be explained by NO synthesis and its immunomodulator effects, especially the antimicrobial properties associated with myristic acid and lauric acid, respectively, which both presented higher levels in CO [44,45,46]. As we mentioned previously, during inflammation, there is an upregulation of adhesion molecules after the venular endothelium is activated and promotes circulating neutrophils to adhere to endothelium sites [47]. Additionally, the production of neutrophils by NO synthases is associated with a reduction in leukocyte recruitment, which exerts anti-inflammatory effects [47]. 

In another study, coconut virgin oil offered protection against free radical formation and reduced inflammation in an arthritic animal model [48]. This model agrees with our experimental model of Carrageenan-induced pleurisy, which represents the main events of acute inflammation in a similar way to that in humans and is considered effective to assess the potential anti-inflammatory action of a compound [49].

Therefore, although some benefits could be attributed to the role of choline, visceral fats, and leukocyte infiltration, CG presented dyslipidemic values for LDL/VLDL and increased glucose and lactate values, which could impact long-term insulin resistance [50]. Thus, prudence is suggested for saturated fatty acid intake as an exclusive or predominant source of lipids, since we do not produce linoleic or linolenic acids, which are thus considered *essentials* and play an important immunomodulating role. Additionally, a deficiency of these acids could contribute to dermatitis problems [51], reenforcing the inclusion of linseed (ALA source).

## 5. Conclusions

In summary, the n-6 acids PUFA and SFA, which are mostly present in SG and CG, did not interact positively in the biochemical analysis (LDL and HDL levels), while LG offered better serum lipid values by increasing HDL-cholesterol and by reducing LDL-cholesterol, as well as triglyceride levels, possibly due to LG’s higher ALA content. Additionally, CG, together with BG, had the lowest adiposity index, and coconut oil presented better immunomodulatory responses with lower leukocyte recruitment. 

The metabolomic assay of the serum samples (NMR data), combined with multi- and univariate statistical analyses, allowed us to determine the metabolic profiles of the *Swiss* mice. With the exception of the baru oil group, the PCA multivariate analysis distinguished the samples and showed important variations in the levels of lactate, LDL/VLDL, and glucose, in which the relationship with coconut oil was evident. 

As discussed in the literature, our study reinforced the controversy regarding the consumption of saturated fatty acids, especially their relationship with the lipid profile. However, coconut oil appears to yield an anti-inflammatory response and reduce body fat, as well as present higher levels of choline, which has lipotropic activities in the liver. Therefore, for future work, a combination of lauric acid and ALA sources is suggested. This combination could produce very effective cardioprotective and anti-inflammatory results.

## Figures and Tables

**Table 1:** Food ingestion, body final weight, weight of liver, and visceral fats in grams (g) based on the dissection and adiposity index for animals treated for 60 days with diets containing different vegetable oils.

Parameter	SG	CG	LG	BG
Food ingestion	5.29 ± 0.64	5.13 ± 0.82	5.27 ± 0.77	5.28 ± 0.69
Body Weight	49.8 ± 7.41	46.72 ± 2.69	47.1 ± 4.39	44.81 ± 5.04
Liver	2.16 ± 0.54	1.97 ± 0.15	1.75 ± 0.24	1.83 ± 0.42
Epididymal	2.54 ± 0.41 ^a^	2.09 ± 0.32 ^a^	2.34 ± 0.52 ^a^	1.40 ± 0.49 ^b,^*
Mesenteric	1.97 ± 0.58 ^a^	1.16 ± 0.21 ^b,†^	1.55 ± 0.28 ^a^	1.08 ± 0.54 ^b,^*
Retroperitoneal	1.11 ± 0.41 ^a^	0.75 ± 0.15 ^a^	0.94 ± 0.22 ^a^	0.61 ± 0.19 ^b,†^
Omental	0.01 ± 0.01	0.01 ± 0.01	0.01 ± 0.01	0.01 ± 0.00
Perirenal	0.64 ± 0.23	0.32 ± 0.06	0.34 ± 0.12	0.24 ± 0.17
Adiposity index (AI)	12.53 ± 2.80 ^a^	9.58 ± 1.02 ^b,^*	11.69 ± 1.67 ^a^	7.32 ± 3.13 ^b,^*

SG: Soybean group; CG: Coconut group; LG: Linseed group; BG: Baru group. Note: Values are expressed as the mean ± SD. Values indicated with different letters in the same line indicate a statistical difference between these groups (*p* < 0.05), while the symbols indicate the value of “*p*”, ^†^ (*p* < 0.01); * (*p* < 0.0001), when compared to the control group (SG); *n* = 12 mice/group, based on one-way ANOVA followed by a Bonferroni test.

**Table 2:** Biochemical parameters of the animals treated with certain diets for 60 days, except for lipid sources, with diets containing different vegetable oils.

Parameter	SG	CG	LG	BG
Blood glucose	147.44 ± 65.01	137.39 ± 50.62	155.24 ± 82.13	98.70 ± 28.05
Total cholesterol	200.03 ± 35.42	208.30 ± 23.75	176.99 ± 19.26	194.96 ± 44.82
HDL-Cholesterol	95.00 ± 28.68 ^a^	47.37 ± 7.47 ^b,†^	76.84 ± 20.06 ^a^	43.35 ± 12.33 ^b,^*
LDL-Cholesterol	131.63 ± 29.03 ^a^	109.35 ± 23.75 ^a^	59.29 ± 7.56 ^b^	103.33 ± 31.72 ^a^
VLDL-Cholesterol	36.63 ± 4.41 ^a^	51.59 ± 14.46 ^b^	40.86 ± 7.80 ^a^	48.29 ± 16.79 ^a^
Non-HDL Cholesterol	131.63 ± 29.03	160.93 ± 22.05	100.15 ± 12.53	148.88 ± 41.80
Triglycerides	183.14 ± 22.06 ^a^	257.93 ± 72.30 ^b^	204.30 ± 38.99 ^a^	241.44 ± 83.93 ^a^

SG: Soybean group; CG: Coconut group; LG: Linseed group; BG: Baru group. Note: Values are expressed as the mean ± SD. Values indicated with different letters in the same line indicate a statistical difference between these groups (*p* < 0.05), while the symbols indicate the value of “*p*”, ^†^ (*p* < 0.01); * (*p* < 0.0001), when compared to the control group (SG); *n* = 12 mice/group, based on one-way ANOVA followed by a Bonferroni test.

## Supplementary Materials

The following supporting information can be downloaded at: https://www.mdpi.com/article/10.3390/nu14061161/s1, Table S1: Fatty acid profile (%) and indices of nutritional quality calculated of prepared diets with soybean, baru, coconut, and linseed oils, Table S2: Quality properties and totals carotenoids of soybean, coconut, linseed, and baru oils, Table S3: Distribution of changes observed on liver tissue of the animals after 60 days of treatment, Table S4: ^1^H (500 MHz) and ^13^C (125 MHz) NMR chemical shifts, multiplicities and coupling constants (J) of the assigned compounds from mouse serum samples fed with animal food containing baru, coconut, linseed, and soybean oils, Figure S1: Induction period of soybean (SO), linseed (LO), coconut (CO) and baru (BO) oils, Figure S2: UV-Vis absorption spectra of vegetable oils diluted at 50% (*v*/*v*) in hexane. Inset shows the absorption spectra collected from high-diluted samples, which the soybean (SO), linseed (LO), coconut (CO) and baru (BO) oils were diluted at 0.13% (*v*/*v*) in hexane, Figure S3: Fluorescence excitation-emission matrix of (a) LO, (b) SO, (c) BO, and (d) CO, Figure S4: Thermal decomposition of BO (baru oil), CO (coconut oil), LO (linseed oil), and SO (soybean oil), Figure S5: Histological analysis of liver tissue from mice. High magnification (400×) photomicrograph, Hematoxylin and eosin stained slided (black arrows indicate macrogoticular steatosis and red arrows indicate hepatocellular ballooning). BG: Baru group; CG: Coconut group; LG: Linseed group; SG: Soybean group. Bar scale: 50 µm, Figure S6. PCA scores (a) and loading (b) plots from 1H NMR spectra (NOESYGPPR1D) of the serum samples from Swiss mice fed diets based on baru, coconut, linseed, and soybean (a,b) and after removal of baru group (c,d). Legend: baru (BG), coconut (CG), linseed (LG), and soybean oil (SG) groups.

## References

[1] Lenighan Y.M., McNulty B.A., Roche H.M. (2019). Dietary Fat Composition: Replacement of Saturated Fatty Acids with PUFA as a Public Health Strategy, with an Emphasis on α-Linolenic Acid. Proc. Nutr. Soc..

[2] Garaulet M., Hernandez-Morante J.J., Lujan J., Tebar F.J., Zamora S. (2006). Relationship between Fat Cell Size and Number and Fatty Acid Composition in Adipose Tissue from Different Fat Depots in Overweight/Obese Humans. Int. J. Obes..

[3] Ibrahim M.M. (2010). Subcutaneous and Visceral Adipose Tissue: Structural and Functional Differences. Obes. Rev..

[4] Yang L.G., Song Z.X., Yin H., Wang Y.Y., Shu G.F., Lu H.X., Wang S.K., Sun G.J. (2016). Low N-6/n-3 PUFA Ratio Improves Lipid Metabolism, Inflammation, Oxidative Stress and Endothelial Function in Rats Using Plant Oils as n-3 Fatty Acid Source. Lipids.

[5] Graham C.B., Philip C.C. Conversion of Alpha-Linolenic Acid to Longer-Chain Polyunsaturated Fatty Acids in Human Adults. https://pubmed.ncbi.nlm.nih.gov/16188209/.

[6] USDA Oilseeds: World Markets and Trade|USDA Foreign Agricultural Service. https://www.ers.usda.gov/data-products/oil-crops-yearbook/oil-crops-yearbook/#World%20Supply%20and%20Use%20of%20Oilseeds%20and%20Oilseed%20Products.

[7] Reis M.Á., Novaes R.D., Baggio S.R., Viana A.L.M., Salles B.C.C., Duarte S.M.D.S., Rodrigues M.R., Paula F.B.D.A. (2018). Hepatoprotective and Antioxidant Activities of Oil from Baru Almonds (Dipteryx Alata Vog.) in a Preclinical Model of Lipotoxicity and Dyslipidemia. Evid.-Based Complement. Altern. Med..

[8] El Riachy M., Hamade A., Ayoub R., Dandachi F., Chalak L. (2019). Oil Content, Fatty Acid and Phenolic Profiles of Some Olive Varieties Growing in Lebanon. Front. Nutr..

[9] Terés S., Barceló-Coblijn G., Benet M., Álvarez R., Bressani R., Halver J.E., Escribá P.V. (2008). Oleic Acid Content Is Responsible for the Reduction in Blood Pressure Induced by Olive Oil. Proc. Natl. Acad. Sci. USA.

[10] Babu A.S., Veluswamy S.K., Arena R., Guazzi M., Lavie C.J. (2014). Virgin Coconut Oil and Its Potential Cardioprotective Effects. Postgrad. Med..

[11] Ulbricht T.L.V., Southgate D.A.T. (1991). Coronary Heart Disease: Seven Dietary Factors. Lancet.

[12] Rodriguez-Amaya D.B., Kimura M. (2004). HarvestPlus Handbook for Carotenoid Analysis.

[13] Latimer J.D.G.W., AOAC (2019). Official Methods of Analysis of AOAC International.

[14] Reeves P.G., Nielsen F.H., Fahey G.C. (1993). AIN-93 Purified Diets for Laboratory Rodents: Final Report of the American Institute of Nutrition Ad Hoc Writing Committee on the Reformulation of the AIN-76A Rodent Diet. J. Nutr..

[15] Taylor B.A., Phillips S.J. (1996). Detection of Obesity QTLs on Mouse Chromosomes 1 and 7 by Selective DNA Pooling. Genomics.

[16] Hagen J.H., Hagen P.B. (1962). An Enzymic Method for the Estimation of Glycerol in Blood and Its Use to Determine the Effect of Noradrenaline on the Concentration of Glycerol in Blood. Can. J. Biochem. Physiol..

[17] Carey R.N., Feldbruegge D., Westgard J.O. (1974). Evaluation of the Adaptation of the Glucose Oxidase/Peroxidase-3-Methyl-2-Benzothiazolinone Hydrazone-N, N-Dimethylaniline Procedure to the Technicon SMA 12/60, and Comparison with Other Automated Methods for Glucose. Clin. Chem..

[18] Flegg H.M. (1973). Ames Award Lecture 1972. An Investigation of the Determination of Serum Cholesterol by an Enzymatic Method. Ann. Clin. Biochem..

[19] de Souza G.E.P., Ferreira S.H. (1985). Blockade by Antimacrophage Serum of the Migration of PMN Neutrophils into the Inflamed Peritoneal Cavity. Agents Actions.

[20] O’Brien R.D. (2008). Fats and Oils: Formulating and Processing for Applications.

[21] SECTION 2 Codex Standards for Fats and Oils from Vegetable Sources. http://www.fao.org/3/y2774e/y2774e04.htm.

[22] Houshia O.J., Zaid O., Shqair H., Zaid M., Fashafsheh N., Bzoor R. (2014). Effect of Olive Oil Adulteration on Peroxide Value, Delta-K and on the Acidity Nabali-Baladi Olive Oil Quality. Adv. Life Sci..

[23] De Oliveira I.P., Correa W.A., Neves P.V., Silva P.V.B., Lescano C.H., Michels F.S., Passos W.E., Muzzi R.M., Oliveira S.L., Caires A.R.L. (2017). Optical Analysis of the Oils Obtained from Acrocomia Aculeata (Jacq.) Lodd: Mapping Absorption-Emission Profiles in an Induced Oxidation Process. Photonics.

[24] Magalhães K.F., Caires A.R.L., Silva M.S., Alcantara G.B., Oliveira S.L. (2014). Endogenous Fluorescence of Biodiesel and Products Thereof: Investigation of the Molecules Responsible for This Effect. Fuel.

[25] Figueiredo P.S., Candido C.J., Jaques J.A., Nunes Â.A., Caires A.R., Michels F.S., Almeida J.A., Filiú W.F., Hiane P.A., Nascimento V.A. (2017). Oxidative Stability of Sesame and Flaxseed Oils and Their Effects on Morphometric and Biochemical Parameters in an Animal Model. J. Sci. Food Agric..

[26] Melo E., Michels F., Arakaki D., Lima N., Gonçalves D., Cavalheiro L., Oliveira L., Caires A., Hiane P., Nascimento V. (2019). First Study on the Oxidative Stability and Elemental Analysis of Babassu (Attalea Speciosa) Edible Oil Produced in Brazil Using a Domestic Extraction Machine. Molecules.

[27] Garcia C.C., Franco P.I.B.M., Zuppa T.O., Antoniosi Filho N.R., Leles M.I.G. (2007). Thermal Stability Studies of Some Cerrado Plant Oils. J. Therm. Anal. Calorim..

[28] Gerbaix M., Metz L., Ringot E., Courteix D. (2010). Visceral Fat Mass Determination in Rodent: Validation of Dual-Energy x-Ray Absorptiometry and Anthropometric Techniques in Fat and Lean Rats. Lipids Health Dis..

[29] Degirolamo C., Shelness G.S., Rudel L.L. (2009). LDL Cholesteryl Oleate as a Predictor for Atherosclerosis: Evidence from Human and Animal Studies on Dietary Fat. J. Lipid Res..

[30] Yanai H., Katsuyama H., Hamasaki H., Abe S., Tada N., Sako A. (2015). Effects of Dietary Fat Intake on HDL Metabolism. J. Clin. Med. Res..

[31] Lottering R.T., Govender M., Peerbhay K., Lottering S. (2020). Comparing Partial Least Squares (PLS) Discriminant Analysis and Sparse PLS Discriminant Analysis in Detecting and Mapping Solanum Mauritianum in Commercial Forest Plantations Using Image Texture. ISPRS J. Photogramm. Remote Sens..

[32] Bernhard W., Poets C., Franz A. (2019). Choline and Choline-Related Nutrients in Regular and Preterm Infant Growth. Eur. J. Nutr..

[33] Abdelhamid A.S., Martin N., Bridges C., Brainard J.S., Wang X., Brown T.J., Hanson S., Jimoh O.F., Ajabnoor S.M., Deane K.H. (2018). Polyunsaturated Fatty Acids for the Primary and Secondary Prevention of Cardiovascular Disease. Cochrane Database Syst. Rev..

[34] Dayrit F.M. (2014). Lauric Acid Is a Medium-Chain Fatty Acid, Coconut Oil Is a Medium-Chain Triglyceride. Philipp. J. Sci..

[35] Deol P., Evans J.R., Dhahbi J., Chellappa K., Han D.S., Spindler S., Sladek F.M. (2015). Soybean Oil Is More Obesogenic and Diabetogenic than Coconut Oil and Fructose in Mouse: Potential Role for the Liver. PLoS ONE.

[36] Tsartsou E., Proutsos N., Castanas E., Kampa M. (2019). Network Meta-Analysis of Metabolic Effects of Olive-Oil in Humans Shows the Importance of Olive Oil Consumption With Moderate Polyphenol Levels as Part of the Mediterranean Diet. Front. Nutr..

[37] Silva É., Spontoni B., Santo E., Paniago G., Portugal L., Dos D., Freitas S., Alcantara G., Filiú W., Freitas K. (2017). Metabolic evaluation of the effects of a hyperlipid diet for obesity induction and standard normolipid diet (ain 93) consumption in wistar rats. Int. J. Dev. Res..

[38] Schenkel L.C., Sivanesan S., Zhang J., Wuyts B., Taylor A., Verbrugghe A., Bakovic M. (2015). Choline Supplementation Restores Substrate Balance and Alleviates Complications of Pcyt2 Deficiency. J. Nutr. Biochem..

[39] Zeisel S.H., da Costa K.-A. (2009). Choline: An Essential Nutrient for Public Health. Nutr. Rev..

[40] Vinegar R., Truax J.F., Selph J.L. (1973). Some Quantitative Temporal Characteristics of Carrageenin-Induced Pleurisy in the Rat. Proc. Soc. Exp. Biol. Med..

[41] Vinegar R., Truax J.F., Selph J.L., Voelker F.A. (1982). Pathway of Onset, Development, and Decay of Carrageenan Pleurisy in the Rat. Fed. Proc..

[42] Rankin J.A. (2004). Biological Mediators of Acute Inflammation. AACN Clin. Issues.

[43] Wong C.H.Y., Heit B., Kubes P. (2010). Molecular Regulators of Leucocyte Chemotaxis during Inflammation. Cardiovasc. Res..

[44] Zhu W., Smart E.J. (2013). Myristic Acid Stimulates Endothelial Nitric-Oxide Synthase in a CD36- and an AMP Kinase-Dependent Manner. J. Biol. Chem..

[45] Alves N.F.B., de Queiroz T.M., de Almeida Travassos R., Magnani M., de Andrade Braga V. (2017). Acute Treatment with Lauric Acid Reduces Blood Pressure and Oxidative Stress in Spontaneously Hypertensive Rats. Basic Clin. Pharmacol. Toxicol..

[46] Angeles-Agdeppa I., Nacis J.S., Capanzana M.V., Dayrit F.M., Tanda K.V. (2021). Virgin Coconut Oil Is Effective in Lowering C-Reactive Protein Levels among Suspect and Probable Cases of COVID-19. J. Funct. Foods.

[47] Jädert C., Petersson J., Massena S., Ahl D., Grapensparr L., Holm L., Lundberg J.O., Phillipson M. (2012). Decreased Leukocyte Recruitment by Inorganic Nitrate and Nitrite in Microvascular Inflammation and NSAID-Induced Intestinal Injury. Free Radic. Biol. Med..

[48] Vysakh A., Ratheesh M., Rajmohanan T.P., Pramod C., Premlal S., Girish kumar B., Sibi P.I. (2014). Polyphenolics Isolated from Virgin Coconut Oil Inhibits Adjuvant Induced Arthritis in Rats through Antioxidant and Anti-Inflammatory Action. Int. Immunopharmacol..

[49] Jantz M.A., Antony V.B. (2008). Pathophysiology of the Pleura. Respiration.

[50] Gao C.-L., Zhu C., Zhao Y.-P., Chen X.-H., Ji C.-B., Zhang C.-M., Zhu J.-G., Xia Z.-K., Tong M.-L., Guo X.-R. (2010). Mitochondrial Dysfunction Is Induced by High Levels of Glucose and Free Fatty Acids in 3T3-L1 Adipocytes. Mol. Cell. Endocrinol..

[51] Lima R.D.S., Block J.M. (2019). Coconut Oil: What Do We Really Know about It so Far?. Food Qual. Saf..

