# Supplementary material for: Linseed, Baru, and Coconut Oils: NMR-Based Metabolomics, Leukocyte Infiltration Potential In Vivo, and Their Oil Characterization. Are There Still Controversies?
Source: Nutrients. 2022 Mar 10;14(6):1161. doi: 10.3390/nu14061161 (PMC8950264; doi:10.3390/nu14061161)
Supplement: Supplementary file 1 [file nutrients-14-01161-s001.zip › nutrients-1597838-supplementary.pdf]

Linseed, baru and coconut oils: NMR-based metabolomics, leukocyte infiltration potential *in vivo*, and their oil characterization. Are there still controversies?

Priscila Silva Figueiredo[1*], Taynara Nogueira Martins[2], Luciana Marçal Ravaglia[2], Glaucia Braz Alcantara[2], Rita de Cássia Avellaneda Guimarães[1], Karine de Cássia Freitas[1], Ângela Alves Nunes[3], Lincoln Carlos Silva de Oliveira[2], Mário Rodrigues Cortês[2], Flavio Santana Michels[4], Mônica Cristina Toffoli Kadri[5], Iluska Senna Bonfá[5], Wander Fernando de Oliveira Filiú[5], Marcel Arakaki Asato[6], Bernardo Bacelar de Faria[6], Valter Aragão do Nascimento[1] and Priscila Aiko Hiane[1]

Supplementary Materials:

Supplemantary Tables:

Table S1. Fatty acid profile (%) and indices of nutritional quality calculated of prepared diets with soybean, baru, coconut, and linseed oils.

Parameters	SO	BO	CO	LO
Fatty acid profile (%)				
Capric, C10:0	0.11±0.02	0.20±0.03	5.80±0.02	0.0±0.00
Lauric, C12:0	0.05±0.01	0.11±0.02	30.16±0.02	0.0±0.00
Myristic, C14:0	0.22±0.03	0.03±0.04	22.13±0.03	0.01±0.00
Palmitic, C16:0	15.04±0.01	8.15±0.01	12.15±0.01	6.02±0.01
Palmitoleic, C16:1	0.13±0.04	0.05±0.00	5.7±0.04	0.33±0.01
Heptadecanoic, C17:0	0.09±0.02	0.07±0.02	4.3±0.02	0.0±0.00
Stearic, C18:0	8.12±0.01	5.13±0.04	3.7±0.01	4.56±0.02
Oleic, C18:1	25.78±0.02	51.30±0.03	4.12±0.01	22.31±0.05
Linoleic, C18:2	42.06±0.03	29.15±0.01	3.32±0.04	18.09±0.05
Linolenic, C18:3	4.16±0.02	0.10±0.01	1.15±0.02	46.80±0.04
Arachidic, C20:0	0.41±0.00	1.78±0.03	2.31±0.02	0.28±0.01
Behenic, C22:0	0.53±0.01	2.88±0.06	1.06±0.01	0.19±0.04
Lignoceric, C24:0	0.13±0.01	0.09±0.07	2.02±0.01	0.02±0.02
**∑ SFA**	24.70	18.96	83.63	11.05
**∑ MUFA**	25.91	51.35	9.82	22.64
**∑ PUFA**	46.22	29.25	4.47	64.89

Indices of nutritional quality				
Atherogenic index (AI)	0.22	0.10	9.16	0.07
Thrombogenic index (TI)	0.50	0.33	3.79	0.06
Hypocholesterolemic:hypercholesterolemic (HH)	4.72	9.85	0.25	14.46

∑: Sum symbol; SFA: Saturated fatty acids; MUFA: Monounsaturated fatty acids; PUFA: polyunsaturated fatty acids. SO: Soybean oil; BO: Baru oil; CO: coconut oil; LO: linseed oil.

Table S2. Quality properties and totals carotenoids of soybean, coconut, linseed, and baru oils.

Parameter	SO	CO	LO	BO
Carotenoids (μg/g)	15.13 ± 0.62^{b}	1.43 ± 0.18 ^{c*}	24.37 ± 1.12^{a*}	2.35 ± 0.03^{c*}
Acidity index (mg $KOHg^{-1}$)	1.75 ± 0.00^{b}	1.01 ± 0.19^{b}	23.67 ± 0.76^{a*}	1.31 ± 0.21^{b}
Peroxide index (meqO_2 kg^{-1})	7.90 ± 0.09^{b}	2.65± 0.00^{c}	7.27 ± 0.92^{b}	15.77 ± 0.17^{a}
Saponification index (mg KOH g^{-1})	187.85 ± 4.88^{b}	247.04 ± 3.78^{a*}	199.08 ± 0.56^{b}	189.90 ± 10.36^{b}
Iodine index (g I_2 100^{-1} g)	150.66±2.33^{a}	23.62±3.49^{b*}	157.90±6.92^{a}	115.20±0.28^{a}
Refraction index at 20 °C	1.473 ± 0.00^{a}	1.456 ± 0.00^{b*}	1.477 ± 0.00^{b†}	1.469 ± 0.00^{b†}

SO: soybean oil; BO: Baru oil; CO: coconut oil; LO: linseed oil. Note: Values expressed as mean ± SD. Different letters in the same line represent statistical differences ($p < 0.05$), being that † represent a very significant difference ($p<0.01$) and * represent a strong significant difference statistical ($p<0.0001$) by one-way ANOVA followed by Tukey test.

Table S3. Distribution of changes observed on liver tissue of the animals after 60 days of treatment.

Variables	SG	CG	LG	BG	p value(a)
Apoptosis					
Absent	91.7 (11)	75.0 (9)	75.0 (9)	83.3 (10)	0,68
Focal (rare)	8.3 (1)	25.0 (3)	25.0 (3)	16.7 (2)	
Ballooning					
Absent	25.0 (3)	16.7 (2)	66.7 (8)	33.3 (4)	0,06
Present	75.0 (9)	83.3 (10)	33.3 (4)	66.7 (8)	
Few cells(b)	33.3 (4)	83.3 (10)	25.0 (3)	50.0 (6)	
Many cells(b)	41.7 (5)	0.0 (0)	8.3 (1)	16.7 (2)	
Steatosis					
< 5	66.7 (8)	83.3 (10)	75.0 (9)	75.0 (9)	0,94
≥ 5	33.3 (4)	25.0 (3)	25.0 (3)	25.0 (3)	
5-33(b)	25.0 (3)	0.0 (0)	16.7 (2)	16.7 (2)	
>33-66(b)	8.3 (1)	16.7 (2)	8.3 (1)	8.3 (1)	
Microvesicular steatosis					
Absent	75.0 (9)(B)	100.0 (12) (A)	91.7 (11) (A)	100.0 (12)(A)	0,03*
Present	25.0 (3)	0.0 (0)	0.0 (0)	0.0 (0)	
No observations(b)	0.0 (0)	0.0 (0)	8.3 (1)	0.0 (0)	
Nuclear glycogenation					
Absent	100.0 (12)	100.0 (12)	100.0 (12)	100.0 (12)	-
Mallory hyaline					
Absent	66.7 (8)	83.3 (10)	91.7 (11)	66.7 (8)	0,34
Present	33.3 (2)	8.3 (1)	8.3 (1)	33.3 (4)	
No observations (b)	0.0 (0)	8.3 (1)	0.0 (0)	0.0 (0)	
Lobular inflammation					
Absent	100.0 (12)	66.7 (8)	50.0 (6)	75.0 (9)	0.05

Present	0.0 (0)	33.3 (4)	50.0 (6)	25.0 (3)	
<2 focus(b)	0.0 (0)	25.0 (3)	50.0 (6)	16.7 (2)	
2-4 focus (b)	0.0 (0)	8.3 (1)	0.0 (0)	8.3 (1)	
Steatosis localization					
Zone 3 or zone 1	50,0 (6)	25.0 (3)	33.3 (4)	33.3 (4)	0.59
Panacinar	8.3 (1)	8.3 (1)	0.0 (0)	0.0 (0)	
No observations (b)	41.7 (5)	66.7 (8)	66.7 (8)	66.7 (8)	

SG: Soybean group; CG: Coconut group; LG: Linseed group; BG: Baru group. (a) Chi-square test with Bonferroni correction; (b) Values not considered in the statistical analysis; In Mallory's Hyaline variable, among those present, only the control group presented several observations, the others only rare. Data presented as relative frequency (absolute frequency). *Statistical association.

Table S4. ^1H (500 MHz) and ^{13}C (125 MHz) NMR chemical shifts, multiplicities and coupling constants (J) of the assigned compounds from mouse serum samples fed with animal food containing baru, coconut, linseed, and soybean oils.

Compound	Assignment	1H NMR chemical shift (ppm); multiplicity (J, in Hz)	13C NMR chemical shift (ppm)
Alanine	CH_3	1.48; d (7.2)	18.8
	CH	3.76; q (7.2)	53.2
β-hidroxibutirate	CH_3	1.20; d (6.3)	24.4
	CH_2	2.31; dd (6.5 and 14.3)	49.2
		2.42; dd (7.3 and 14.3)	49.2
	CH	4.16; m	68.4
Lactate	CH_3	1.34**; d (6.9)	22.6
	CH	4.12; q (6.9)	71.2
α-glucose	C-1H	5.24**; d (3.8)	94.6
β-glucose	C-1H	4.65**; d (7.9)	98.4
Citrate	α,γ-CH	2.53; d (15.5)	48.1
	α',γ'-CH	2.67; d (15.5)	48.1
Acetate	CH_3	1.92; s	25.8
Creatine/creatinine	N-CH_3	3.04**; s	39.1 and 28.3
	N-CH_2	3.92; s	56.5 ande 58.2
LDL/VLDL	CH_2	1.27; bs	-
	CH_3	0.87**; bs	-
Lipids	$(CH_2)_n$	1.30; bs	-
	CH_2-CH_2-CO	1.57; bs	-
	CH_2-CH_2-CO	2.24; bs	-
	unidentified[a]	4.06; m	64.4
	unidentified[a]	4.27; m	64.4
	unidentified	2.85; m	-
	CH=	5.3**; sl	-

Unsaturated fatty acids	-CH=CH-CH_2-	2.02; sl	-
	=CH-CH_2-CH=	2.76; m	-
Leucine	β-CH	1.72	42.7
	δ-CH_3	0.95; m	26.2
	N-CH	3.75; m	51.1
Valine	CH_3	1.05; d (7.1)	19.4
	CH_3	0.99; d (7.1)	20.4
Glutamine/glutamate	N-CH	3.76; dd (5.4 and 12.3)	56.7
	CH_2	2.14; m	29.2
	CH_2	2.43; dd (7.3 and 14.3)	33.7
Succinate	CH_2	2.40; s	40.0
Choline	N-$(CH_3)_3$	3.21**; s	56.2
Phosphocholine	N-$(CH_3)_3$	3.23; s	56.4
	O-CH_2	4.33; m	62.0
Taurine	CH_2	3.27; t (6.5)	50.4
	N-CH_2	3.42; t (6.5)	37.7
Tyrosine	CH(2,6)	6.89; d (8.5)	-
	CH(3,5)	7.19; d (8.5)	-
Histidine	C2H	7.86	-
	C5H	7.09	-
Phenylalanine	C2H,C6H	7.32	-
	C3H,C5H	7.42	-
	C4H	7.38	-
Tryptophan	C7H	7.53	-
	C4H	7.73	-
	C6H	7.25	-
Formate	HCOO-	8.45; s	-

*s: singlet, bs: broad singlet, d: doublet, t: triplet, q: quartet, m: multiplet.

**: signal used for the relative proportion determination.

[a] unidentified (probably CH_2 signal from triacylglycerol).

Supplemantary Figures:

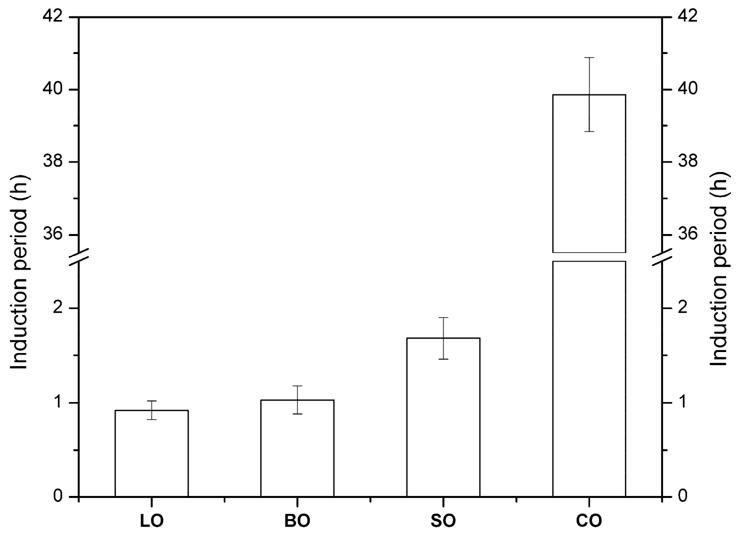

Figure S1. Induction period of soybean (SO), linseed (LO), coconut (CO) and baru (BO) oils.

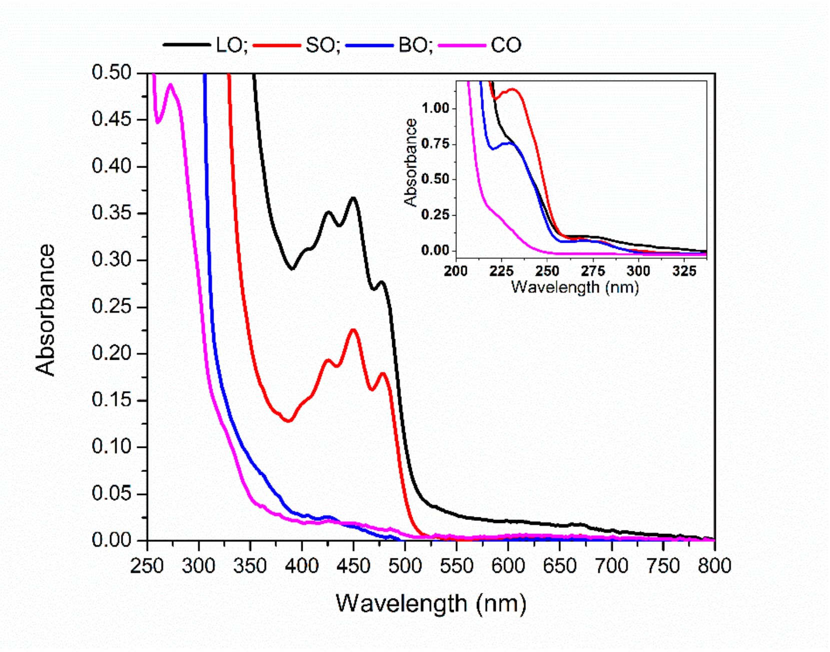

Figure S2. UV-Vis absorption spectra of vegetable oils diluted at 50% (v/v) in hexane. Inset shows the absorption spectra collected from high-diluted samples, which the soybean (SO), linseed (LO), coconut (CO) and baru (BO) oils were diluted at 0.13% (v/v) in hexane.

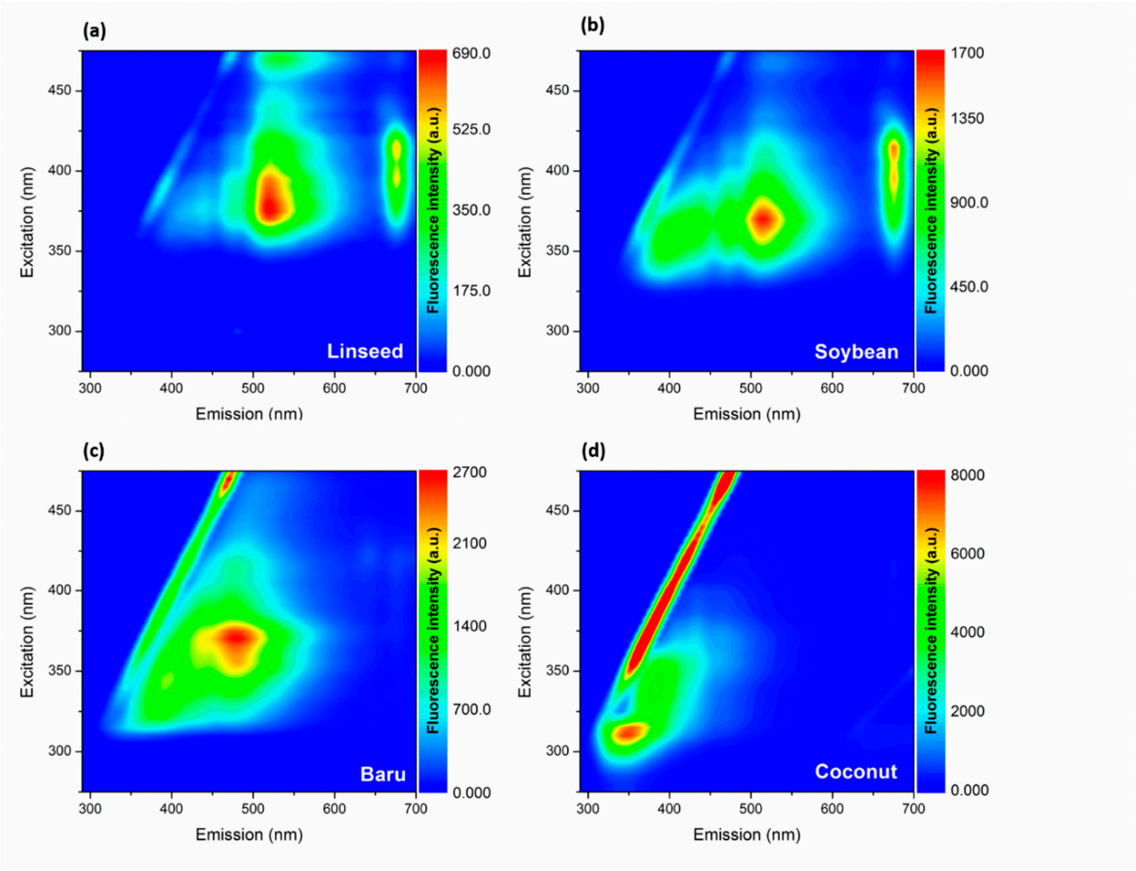

Figure S3. Fluorescence excitation-emission matrix of (a) LO, (b) SO, (c) BO, and (d) CO.

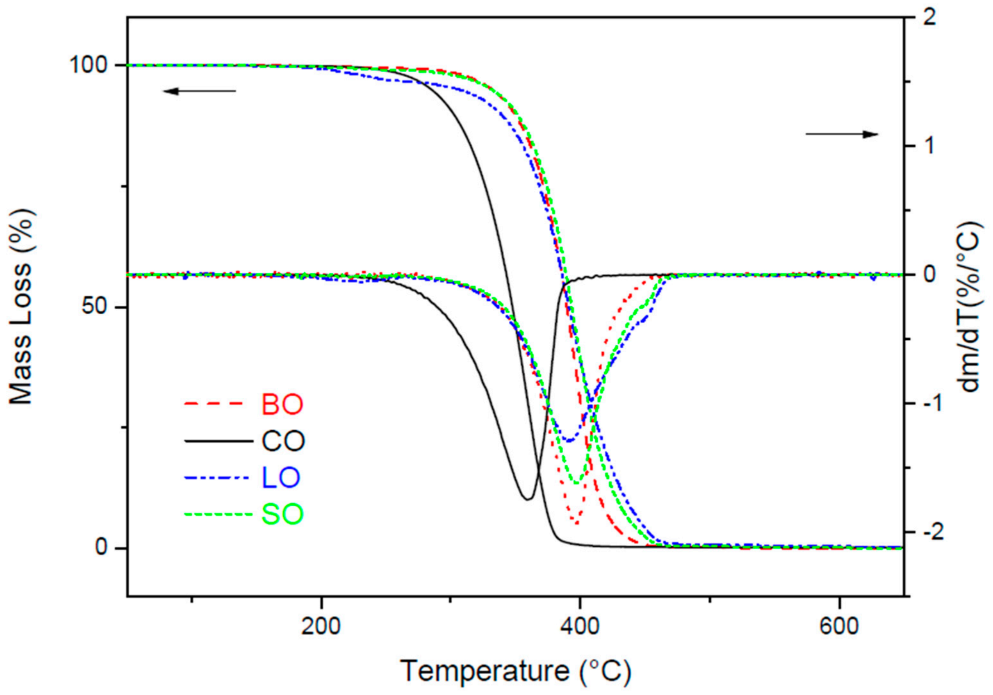

Figure S4. Thermal decomposition of BO (baru oil), CO (coconut oil), LO (linseed oil), and SO (soybean oil).

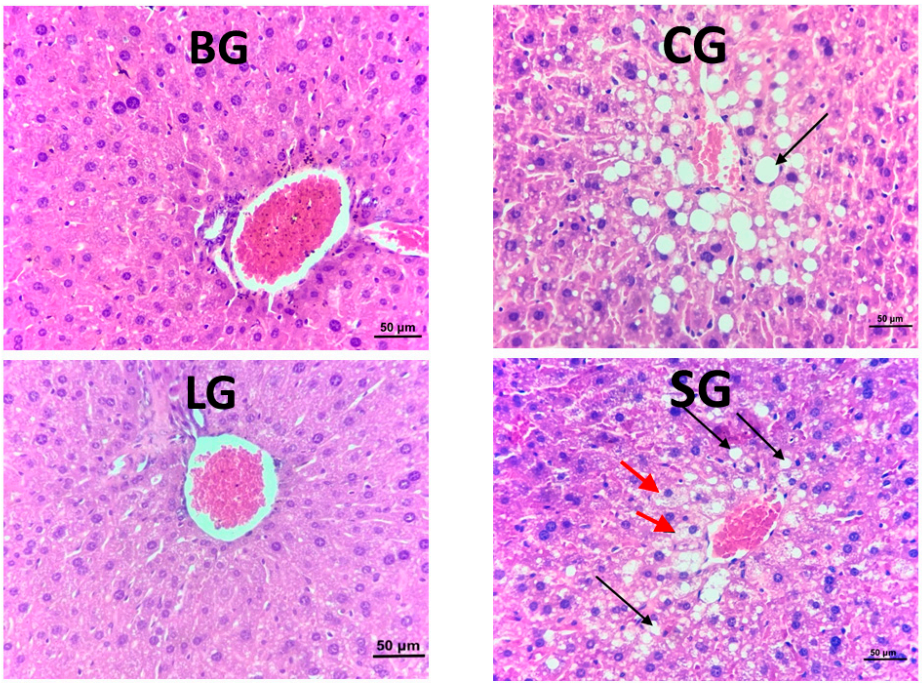

Figure S5. Histological analysis of liver tissue from mice. High magnification (400x) photomicrograph, Hematoxylin and eosin stained slided (black arrows indicate macrogoticular steatosis and red arrows indicate hepatocellular ballooning). BG: Baru group; CG: Coconut group; LG: Linseed group; SG: Soybean group. Bar scale: 50µm.

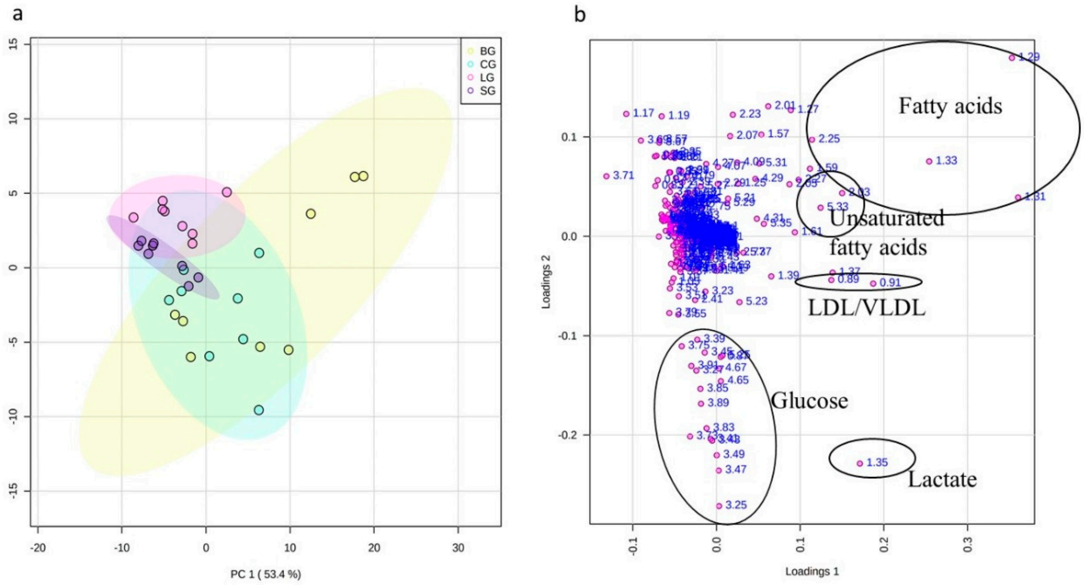

Figure S6. PCA scores (a) and loading (b) plots from ^{1}H NMR spectra (NOESYGPPR1D) of the serum samples from Swiss mice fed diets based on baru, coconut, linseed, and

soybean (a,b) and after removal of baru group (c,d) . Legend: baru (BG), coconut (CG), linseed (LG), and soybean oil (SG) groups.
